# Numerical investigation of the elastic scattering of hydrogen (isotopes) and helium at graphite (0001) surfaces at beam energies of 1 to 4 eV using a split-step Fourier method

**DOI:** 10.1007/s00214-013-1337-9

**Published:** 2013-02-07

**Authors:** Stefan E. Huber, Tobias Hell, Michael Probst, Alexander Ostermann

**Affiliations:** 1 Institute for Ion Physics and Applied Physics, Innsbruck University, Technikerstrasse 25, 6020 Innsbruck, Austria; 2 Department of Mathematics, Innsbruck University, Technikerstrasse 13, 6020 Innsbruck, Austria

**Keywords:** Surface scattering, DFT, Splitting method, Magnetic fusion, Plasma-wall interaction, Time-dependent wave packet simulation

## Abstract

We report simulations of the elastic scattering of atomic hydrogen isotopes and helium beams from graphite (0001) surfaces in an energy range of 1–4 eV. To this aim, we numerically solve a time-dependent Schrödinger equation using a split-step Fourier method. The hydrogen- and helium-graphite potentials are derived from density functional theory calculations using a cluster model for the graphite surface. We observe that the elastic interaction of tritium and helium with graphite differs fundamentally. Whereas the wave packets in the helium beam are directed to the centers of the aromatic cycles constituting the hexagonal graphite lattice, they are directed toward the rings in case of the hydrogen beams. These observations emphasize the importance of swift chemical sputtering for the chemical erosion of graphite and provide a fundamental justification of the graphite peeling mechanism observed in molecular dynamics studies. Our investigations imply that wave packet studies, complementary to classical atomistic molecular dynamics simulations open another angle to the microscopic view on the physics underlying the sputtering of graphite exposed to hot plasma.

**Electronic supplementary material:**

The online version of this article (doi:10.1007/s00214-013-1337-9) contains supplementary material, which is available to authorized users.

## Introduction

The divertor in the next step nuclear fusion device ITER is planned to be shielded against hot hydrogen plasma by plasma facing component (PFC) materials, in some scenarios carbon (target plates) and tungsten (upper divertor and dome) [1]. The choice of graphite (carbon) PFC materials has been made based on its high thermal conductivity, thermo-mechanical resistivity and the fact that it does not melt but only sublimates [2]. Major advantages of tungsten over carbon are its lower erosion rates and thus its longer lifetime. Its limitations are related to its thermal behavior under high off-normal heat loads like they might occur in large edge-localized modes [3, 4] and its stiffness. One of the major disadvantages of carbon is its high chemical reactivity with hydrogen (and/or isotopes) leading to erosion processes summarized under the expression chemical erosion [4–7]. This drastically limits the utilization of carbon-based materials due to safety concerns. In fact, it is planned to replace the carbon-based divertor plates with a full tungsten divertor at least in a later stage of the fusion experiment ITER [1]. The use of tungsten blocks below their ductile to brittle transition temperature could result in the total failure through the cracking of tungsten near cooling tubes [8]. Hence, carbon-based materials should at least be kept in mind as an alternative for PFC materials in ITER and maybe even for reactors [8, 9]. Fusion experiments (for an overview, see for instance [4, 7, 10]) imply that carbon is significantly eroded by incoming hydrogen isotopes due to the sputtering of hydrocarbon molecules already at low plasma temperatures of about 1–10 eV. To complement these experiments as well as to provide an atomistic view on the chemical erosion of carbon-based materials, a variety of molecular dynamics (MD) simulations have been carried out [11–25]. These studies reveal several mechanisms that are important for the chemical erosion of carbon-based materials, of which three phenomena are of particular interest for the scope of this work. First, swift chemical sputtering has been proposed [11] as one of the main mechanisms leading to significant erosion at low impact energies. The sputtering proceeds as impinging hydrogen atoms penetrate the region between carbon–carbon bonds and, if their energies are in a certain range, subsequently break the bonds. Second, MD studies show that the addition of noble gases in small concentrations (less than 10 per cent) to the usual impact species hydrogen (isotopes) does not significantly alter the erosion yield [12]. Third, MD studies involving pure perfect hexagonal graphite reveal the interesting sputtering mechanism known as graphite peeling, that is as the graphite layers are bombarded they are peeled off one after the other [21].

In contrast to classical MD simulations, we treat elastic collisions of H/D/T (hydrogen/deuterium/tritium) and He with a graphite (0001) surface quantum-mechanically. For this purpose, we derive H/D/T-graphite and He-graphite potentials from quantum chemical cluster calculations and model the H/D/T and He nuclei as appropriate wave packets. The time-dependent Schrödinger equation is solved using a split-step Fourier method. This numerical approach has already proven to be beneficial in a variety of applications such as thermal energy atomic scattering or molecular beam scattering [26–29]. For example, in [30] elastic scattering of low energy He beams in the range of 10^−2^ eV with a rigid monolayer of xenon atoms was simulated in good agreement with experiment. More relevant in terms of nuclear fusion and the present work has been a wave packet study investigating the interaction of H and D with the basal plane of graphite in a range of impact energies of 0.1–0.9 eV [31]. In this work, we deal with beam energies in the range of 1–4 eV, which are substantially higher than the ones in these earlier applications, and therefore, they are believed to be of significant relevance for fusion research in the field of plasma-wall interaction. Though only elastic scattering is studied, our results are in agreement with the physics underlying the three previously described phenomena revealed by earlier MD studies and the mentioned wave packet study.

In Sect. 2, the numerical method is presented and the construction of the H/D/T-graphite and the He-graphite potentials is described. The results are discussed in Sect. 3, focusing especially on the significant difference between H/D/T and He scattering. Finally, in Sect. 4, a conclusion is given.

## Methodology

We briefly describe the used numerical scheme, a Fourier split-step method, and the construction of the H/D/T- and He-graphite-potentials which is the input to the propagation method.

### Numerical solution of the Schrödinger equation

In order to model the dynamics of elastic scattering from a quantum mechanical point of view, one has to solve the time-dependent Schrödinger equation, which—using mass-scaled coordinates—reads

1 $$ \begin{gathered} i\partial_{t} \Uppsi \left( {t,x} \right) = - \Updelta \Uppsi \left( {t,x} \right) + V\left( x \right)\Uppsi \left( {t,x} \right),\quad  t > 0,x \in {\mathbb{R}}^{3} , \hfill \\ \Uppsi \left( {0,x} \right) = \Uppsi_{0} \left( x \right), \quad x \in {\mathbb{R}}^{3} , \hfill \\ \end{gathered} $$

where

$$ \Uppsi :\left[ {0,\infty } \right) \times {\mathbb{R}}^{3} \to {\mathbb{C}} $$

denotes the wave function, *V* is the time-independent potential describing either the hydrogen- or the helium-graphite-potential and ${{\Uppsi}}_{0}$ is a given smooth initial function, in our case a Gaussian wave packet. Note that the original problem can be reformulated in such a way by choosing appropriate units. To numerically approach Eq. (1), we use a so-called splitting method. The basic idea of Lie splitting is to solve the potential part of Eq. (1)

2 $$ i\partial_{t} \Uppsi_{P} \left( {t,x} \right) = V\left( x \right)\Uppsi_{P} (t,x) $$

and to use the respective solution as initial value for the kinetic part

3 $$ i\partial_{t} \Uppsi_{K} \left( {t,x} \right) = - \Updelta \Uppsi_{K} (t,x) , $$

that is the free-particle Schrödinger equation. Given a temporal step size *h* > 0, set *t*_*n*_ = *nh* and $\Uppsi_{n}^{(L)}$ denoting the numerical approximation obtained by the Lie splitting at time *t* = *nh* and using the notion of groups, the resulting method reads as

$$ \Uppsi_{n + 1}^{(L)} = e^{ - ihV} e^{ih\Updelta } \Uppsi_{n}^{(L)} , $$

where $\Uppsi_{0}^{(L)} = \Uppsi_{0}$. Under appropriate assumptions, the method is of order one, that is

$$ {\left\| {\Uppsi_{n}^{(L)} - \Uppsi (nh, \cdot )} \right\|} \le C_{L} h \quad {\text{for}}\, 0 \le nh \le T, $$

where *C*_*L*_ > 0 denotes a constant not depending on the step size *h*.

By symmetrizing the Lie splitting, one obtains the Strang splitting, cf. [32], given by

$$ \Uppsi_{n + 1}^{(S)} = e^{{ - i\frac{h}{2}V}} e^{ih\Updelta } e^{{ - i\frac{h}{2}V}} \Uppsi_{n}^{(S)} , $$

which is of order two, that is

$$ {\left\| {\Uppsi_{n}^{(S)} - \Uppsi (nh, \cdot )} \right\|} \le C_{S} h^{2} \quad   {\text{for}}\, 0 \le nh \le T $$

and some constant *C*_*S*_ > 0 independent of *h*.

By choosing an appropriately large domain, we can artificially impose periodic boundary conditions. As a result, Eq. (3) can be solved very efficiently by using a Fourier spectral method. Note that the other subproblem, Eq. (2), is simply solved by pointwise multiplication. The resulting numerical scheme is known as the split-step Fourier method, cf. [33].

Let $\Uppsi_{n}^{(K)}$ denote the numerical approximation at time *t* = *nh* using $K \in {\mathbb{N}}$ grid points in each space dimension. As described in [33], a single time step is schematically computed as follows:

1. Pointwise multiplication: $$ \Uppsi_{n}^{(K)} := e^{ - ihV/2} \Uppsi_{n}^{(K)} $$
2. FFT: $$ \hat{\Uppsi }_{n}^{(K)} := \mathcal{F}_{K} \Uppsi_{n}^{(K)} $$
3. Multiplication in Fourier space: $\hat{\Uppsi }_{n}^{(K)} := e^{ih\lambda } \hat{\Uppsi }_{n}^{(K)}$
4. IFFT: $$ \Uppsi_{n}^{(K)} := \mathcal{F}_{K}^{ - 1} \hat{\Uppsi }_{n}^{(K)} $$
5. Pointwise multiplication: $$ \Uppsi_{n}^{(K)} := e^{ - ihV/2} \Uppsi_{n}^{(K)} $$

Here, *λ* refers to the respective eigenvalues associated with the Laplacian. Note that one can combine the computation in (1) and (5) if one does not need dense output. For further details, we refer to [34], where also structure-preserving properties of the split-step Fourier method as unitarity, symplecticity and time-reversibility are discussed.

We found this method to perform very well when applied to our problems and since our potential *V* is expressed in quintic smoothing splines, the applied split-step Fourier method is second-order convergent, that is if $\Uppsi_{n}$ denotes the numerical approximation at *t* = *nh*, it holds that

$$ {\left\| {\Uppsi_{n} - \Uppsi (nh)}  \right\|}  \le Ch^{2} \quad  {\text{for}}\, 0 \le nh \le T, $$

where the constant *C* > 0 does not depend on the step size *h*, see [33].

### Construction of the potentials

The determination of energy barriers arising from the permeation of different atoms through models of a graphite (0001) surface is discussed in detail in Ref. [35], and we give only a short summary of the procedure to construct the H/D/T- and He-graphite potentials.

**Fig. 1:**
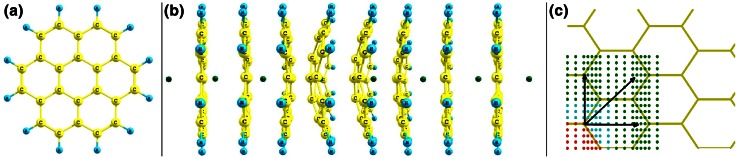
**a** Coronene as a model for the graphite (0001) surface. **b** Permeation of He (*dark green sphere*) through coronene. The PAH is fully relaxed at each step. Geometries are calculated at the B3LYP/6-31G level of theory. **c** Schematics of the construction of the total surface potential by using symmetry. The energy barriers at the locations of the *blue dots* coincide with those calculated at 21 considered permeation sites marked by *red dots*) and periodic continuation (*arrows* and *green dots*) from energy barriers at the 21 permeation sites

The permeation of an atom through a graphite (0001) surface is investigated by quantum chemical cluster calculations in which graphite is modeled by the PAH molecule coronene, see Fig. 1a. PAH molecules are adequate models for graphite surfaces [36] and graphite (0001) surfaces can be interpreted as infinitely sized PAH molecules. Recent studies [35] have shown that the size of coronene (C_24_H_12_) is already sufficient to estimate the potential energy within an accuracy of about 10 % at the barrier maximum. The three-dimensional H/D/T- and He-graphite potentials are constructed in the following way (Fig. 1). First, the distance between H or He and the plane of coronene is varied in 30 steps of 0.15 Å each. Thus, the atom is moved along the *z*-axis toward the molecular plane (the *xy*-plane) of the undisturbed PAH from 3 Å in front to 3 Å behind the PAH molecule (Fig. 1b). This process is performed for 21 distinct permeation sites in a quarter of the central aromatic cycle of coronene. At each position, the energy *E*(A-PAH, *z*) of the total system is calculated after relaxation of the PAH molecule, that is the adiabatic energy barrier *E*(*z*) at the respective position *z* of the contaminant atom is then given as the difference *E*(*z*) = *E*(A-PAH, *z*) − (*E*(A) + *E*(PAH)), where *E*(A) and *E*(PAH) denote the energies of the isolated atom and unperturbed coronene, respectively. Geometries and energies are obtained by density functional theory using the B3LYP functional [37] in conjunction with the small split-valence basis set 6-31G [38]. The appropriateness of this chemical model has been validated in [35]. By comparison with the results obtained with the more sophisticated ωB97XD functional [39] and larger basis sets, it has been found that shortcomings of the B3LYP functional, for example, the neglect of dispersion forces and the basis set truncation error cancel each other. Our model agrees with earlier theoretical [40–43] as well as experimental [44] studies on hydrogen adsorption on graphite: The potential minima are above the carbon atoms, that is at top sites about 1.4 Å from the undisturbed surface. Moreover, the change from sp^2^ to sp^3^ hybridization of the carbon atom where the hydrogen is adsorbed is well reproduced [35]. Quantitatively, however, the accordance is just reasonable. In [44] a desorption energy of 0.7 eV (the energy needed to release an adsorbed hydrogen from the surface) and an activation energy of 0.18 eV are given, whereas our constructed potential yields 0.43 and 0.4 eV, respectively. On average, the accuracy of our model is approximately 0.5 eV. All quantum chemical computations have been carried out with the Gaussian 09 software [45]. By using the symmetry and periodic continuation, the potentials obtained from coronene are extended to model the H/D/T- and He-graphite potential for an infinite surface, see Fig. 1c.

The barrier heights, that is the energies that incoming classical particles need to have at least to penetrate the surface are about 4.5 eV in case of the H/D/T-graphite potential and about 10 eV in case of the He-graphite potential. Since we are only considering elastic reflection processes at energies below these limits, we believe that the approximations introduced by our adiabatic potentials are quite reasonable. Furthermore, it should be noted that the adiabatic approximation is well applicable for the range of projectile energies considered here since the highest energy of 4 eV is much below the HOMO–LUMO gap of coronene (about 9 eV [46]) used to model the graphite (0001) surface.

## Results and discussion

We simulate the elastic scattering of H/D/T- and He beams, at a graphite (0001) surface with the wave packet method described in Sect. 2.1. The hydrogen isotopes are all chemically equivalent and therefore the potentials of H, D and T are the same. For convenience, we focus mainly on tritium for the following reasons. Since the mass of T is most similar to the one of helium, one can treat both elements using the same computational domain as well as spatial resolution. For lighter isotopes, the spatial width of the wave packet is larger and consequently the use of different computational domains becomes necessary to capture the essential features of the scattering process. In particular, the energy width of the beam Δ*E* = 0.2. eV is inversely related to the spatial width via Heisenberg’s uncertainty relation $\Updelta k\Updelta x \ge 1$ and $E = k^{2} /2\;{\text{m}}$, where *k* denotes the momentum of the beam and *m* the particle mass. However, the results for tritium scattering are qualitatively applicable for the lighter hydrogen isotopes too and treating H or D results in a scaling of the temporal and spatial dimensions proportional to a factor of $\sqrt m$ as will be shown below.

For T and He, except for the mass, the same input parameters are used in the simulations. The energies of the particles are 1, 2, 3 and 4 eV, the energy width has been chosen to be 0.2 eV and in all cases the angle of impact is 90°, corresponding to a perpendicular beam direction with respect to the surface. The size of the time steps are 200, 160, 140 and 120 a.u. for the four different beam energies, respectively, leading to 41 time steps in each simulation. The size of the computational domain is 12.5041 × 14.5418 × 18 Å^3^ in which 256 × 256 × 512 grid points define the spatial resolution. The interaction potential is active only in the last third of the computational domain with respect to the *z*-direction, that is in the region 3 Å < *z* < 9 Å, whereas the z-limits are between −9 Å and +9 Å. The interaction sites, that is the regions where the wave packet impinges at the surface, have been chosen to be the three high-symmetry sites of the graphite hexagonal lattice: The hollow site in the center of one aromatic cycle, the bridge site at the center of one C–C bond and the top site directly on the top of one of the carbon atoms. Thus, we have to simulate 24 scattering events in total (four energies and three sites for two nuclei).

One approach to assess the results is to inspect a cut through the computational domain parallel to the surface, the ‘window’ [29], and observe the wave packet as it moves through. The wave packet passes the window twice, that is when it approaches the interaction region and after it is reflected from the surface. The window is placed near the plane of the PAH at *z* = 3 Å.

We start with the discussion of the elastic reflective scattering of He. The He-graphite potential is purely repulsive and does not contain features like, for example, adsorption minima, whereas the H/D/T-graphite potentials do (see Sect. 2.2). Therefore, the physical interpretation is easier and can serve as a basis for the assessment of the more complex situation of H/D/T scattering.

### Elastic scattering of He

**Fig. 2:**
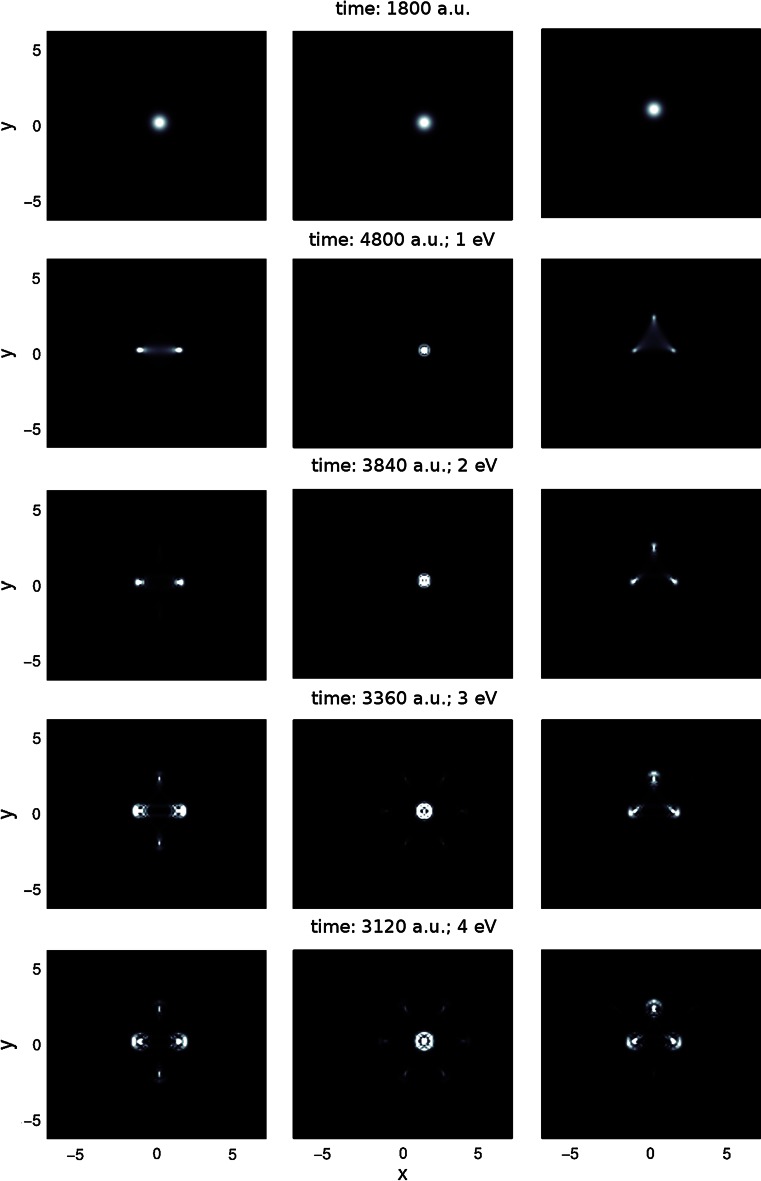
Reflection patterns for He-graphite interaction in the range of 1–4 eV (from *top* to *bottom*) at the three interaction sites bridge (*left*), hollow (*center*) and top (*right*). As reference, the initial wave packets are shown in the *top row*

In Fig. 2, the reflection patterns are depicted for the energy range of 1–4 eV at all three interaction sites (movies illuminating the dynamics of the reflection process are available as supplementary material in the online version of this article). He is deflected from the boundary of the aromatic cycles constituting the graphite lattice. For the impact at bridge site, this means that the initially spherical symmetric He wave packet (top row in Fig. 2) is symmetrically split into two parts by the carbon–carbon bond. At the hollow site, the interaction with the potential valley causes a focusing effect similar to light being reflected by a parabolic mirror. At the top site, the wave packet is symmetrically split into three parts pointing toward the centers of the adjacent hexagonal rings. In addition, for higher energies, the quantum nature of the scattering process becomes visible. Several maxima and minima form in the reflection pattern due to quantum interferences. At the highest energies of 3 and 4 eV, the interaction is strong enough that even far outlying parts of the initial wave packet are focused considerably in hexagonal rings adjacent to the central one, that is six, two and three side maxima are observable besides the strong central feature of the reflection pattern in the case of the hollow, the bridge and the top site, respectively.

**Fig. 3:**
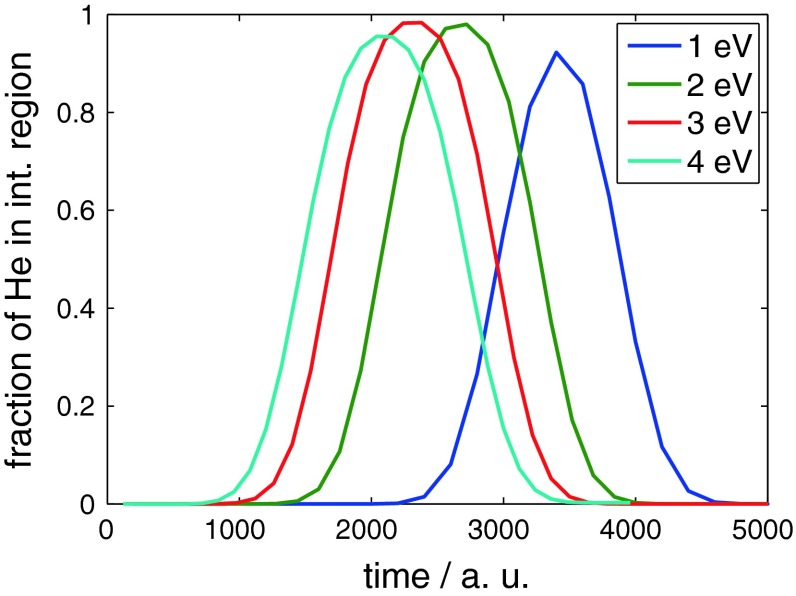
Dwell time in the case of He reflection at graphite (0001) surfaces for different impact energies

The He-graphite potential is purely repulsive and most repulsive at the boundary of the aromatic cycles, that is at the position of the carbon atoms and at carbon–carbon bonds. This can nicely be seen in plots of the dwell time, that is the probability distribution inside a certain interaction region versus time. The interaction region has been chosen as the interval from 4.5 to 9 Å in the computational domain. The part of the system beyond the potential maximum is never reached at the considered energies, and thus, the actual interaction region ranges from 1.5 Å in front of the undisturbed surface to the turning point of the wave packet. For purely repulsive interaction, the dwell time exhibits a Gaussian shape since no partial reflection occurs on the way back of the wave packet due to the monotone shape of the potential. This is the case at all energies as can be seen in Fig. 3. In addition, no difference between the distinct impact sites is observed. The different maxima of the dwell time curves for the individual energies simply refer to the fact that at low energies not the whole wave packet is able to penetrate the interaction region, whereas at high energies parts of the wave packet have already left the interaction region as recently as other parts are not there yet. The shift of the dwell time curves to the left with increasing energy results simply from the fact that all wave packets have the same starting location at 6 Å in front of the undisturbed surface.

**Fig. 4:**
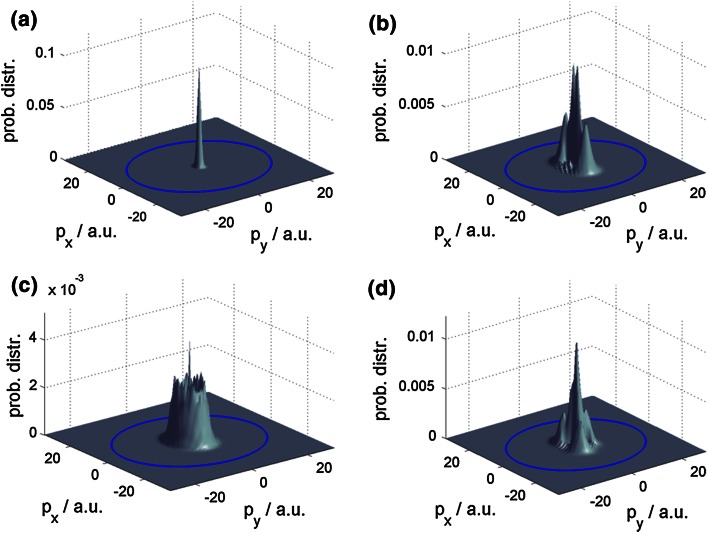
Projection of the probability distribution in momentum space for an impact energy of 1 eV. **a** Initial wave packet. **b** Final probability distribution after reflection at the bridge site. **c** Final distribution after reflection at the hollow site. **d** Final distribution after reflection at the *top* site. Ewald’s circle is indicated as a *blue line*

Additional information can be gathered from projecting the probability distribution in the momentum space into *p*_*x*_ and *p*_*y*_. In Fig. 4, this is done for the initial wave packet (away from the interaction zone) as well as for the final reflected beam (again away from the interaction zone) for the three different interaction sites for 1 eV. Initially, the wave packet corresponds to a peak at the origin of the *xy*-plane with a width corresponding to the energy width of 0.2 eV as discussed at the beginning of Sect. 3. After reflection at the bridge site, four main scattering channels symmetrically shifted to two opposite sites from the origin are observed, in accordance with the direct inspection of the reflection patterns. Analogously, for an impact at the top site, one observes a partial shift of the central peak to three main peaks separated from each other by an angle of 120° in accordance with the broad diffuse zone between the three main peaks in Fig. 2. For impact at the hollow site, the central peak becomes tighter and is symmetrically encircled by a ring-like distribution, again corresponding well to the same feature in Fig. 2. The projection of the probability distribution in momentum space has the advantage that, due to energy conservation, the angular dependence of the reflection pattern can be inspected simply by plotting Ewald’s circle referring to the initial momentum of the wave packet. The part of the probability distribution located at the origin then refers to a reflection perpendicular to the surface, that is a reflection angle of 0°, whereas toward Ewald’s circle the reflection angle increases and on it corresponds to a reflection angle of 90°, that is, parallel to the surface.

### Elastic scattering of H/D/T

**Fig. 5:**
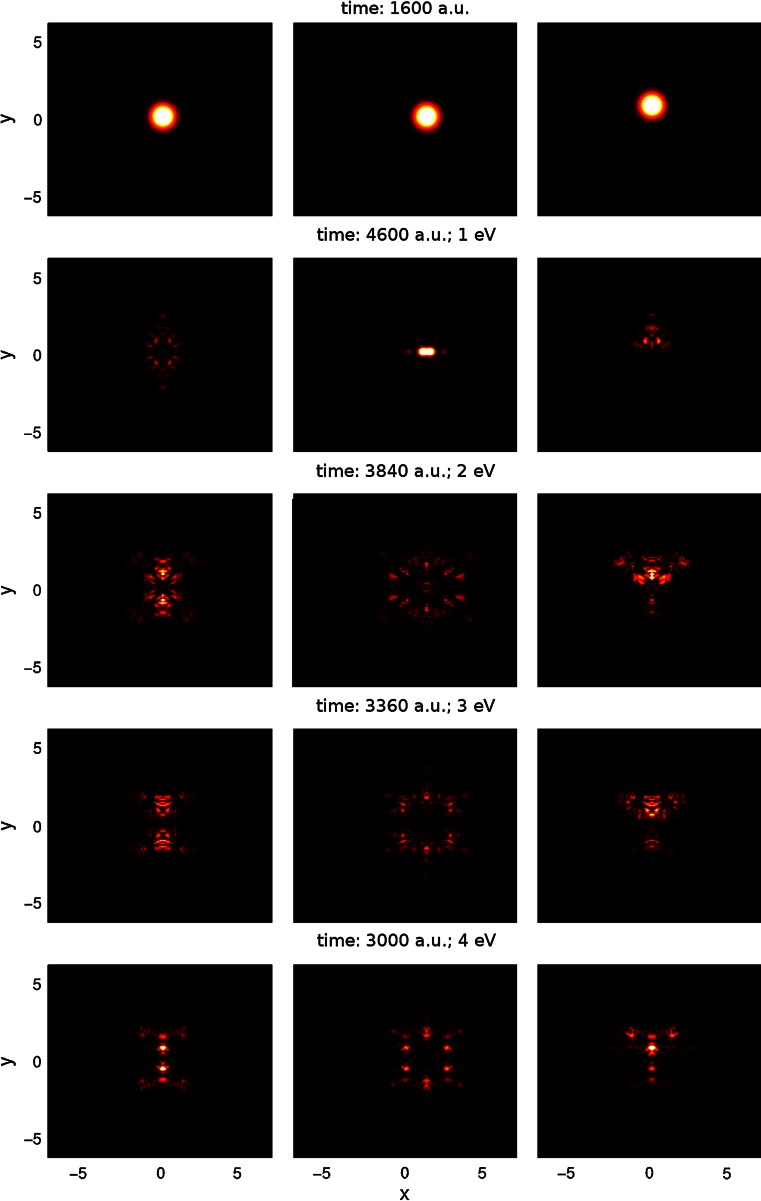
Reflection patterns for the T-graphite interaction in the range of 1–4 eV (from *top* to *bottom*) at the three interaction sites bridge (*left*), hollow (*center*) and top (*right*). As reference, the initial wave packets are shown in the *top row*

In Fig. 5, the reflection patterns are depicted for the energy range of 1–4 eV at all three interaction sites (movies illuminating the dynamics of the reflection process are available as supplementary material in the online version of this article). The results exhibit more complexity than in the case of He scattering and a stronger energy dependence. For 1 eV one recognizes similar features as in the case of He scattering. However, already at this low impact energy, some differences are apparent. At the bridge site, the wave packet appears to be deflected by the carbon–carbon bond but exhibits much more structure due to interference. The initially spherical wave packet is distributed into an ellipsoid-like shape around the region of the bond and is not separated into two distinct wave packets at the two sides of the bond. At the hollow site, the wave packet is not focused but rather smeared out, pointing toward the edges of the aromatic ring. Inspection of the reflection patterns at different locations of the window reveals that in case of the hollow site, the wave packet is mainly split into two parts which are deflected toward the carbon–carbon bonds on the left and right of the hexagon, see Fig. 5. For the top site, one observes a similar triangular shape of the reflection pattern as for He, but again, it is smeared out and does not yield three well-separated reflection peaks but is distributed rather around the carbon atom. At 2–4 eV, the situation becomes clearer. It turns out that the T wave packet is attracted by the boundary of the hexagonal aromatic cycles that constitute the graphite surface. This can be seen best in the reflection patterns for the hollow site, where at the starting energy of 2 eV a hexagonal structure in the reflection pattern becomes observable. The higher the energy, the more concise and compact the structure of the reflection pattern becomes. In addition, parts of the adjacent aromatic cycles can be observed in it. The same is true for the bridge and the top sites. Altogether, the T wave packet is rather deflected away from the centers of the aromatic cycles and attracted to their boundary in the energy range of 2–4 eV.

**Fig. 6:**
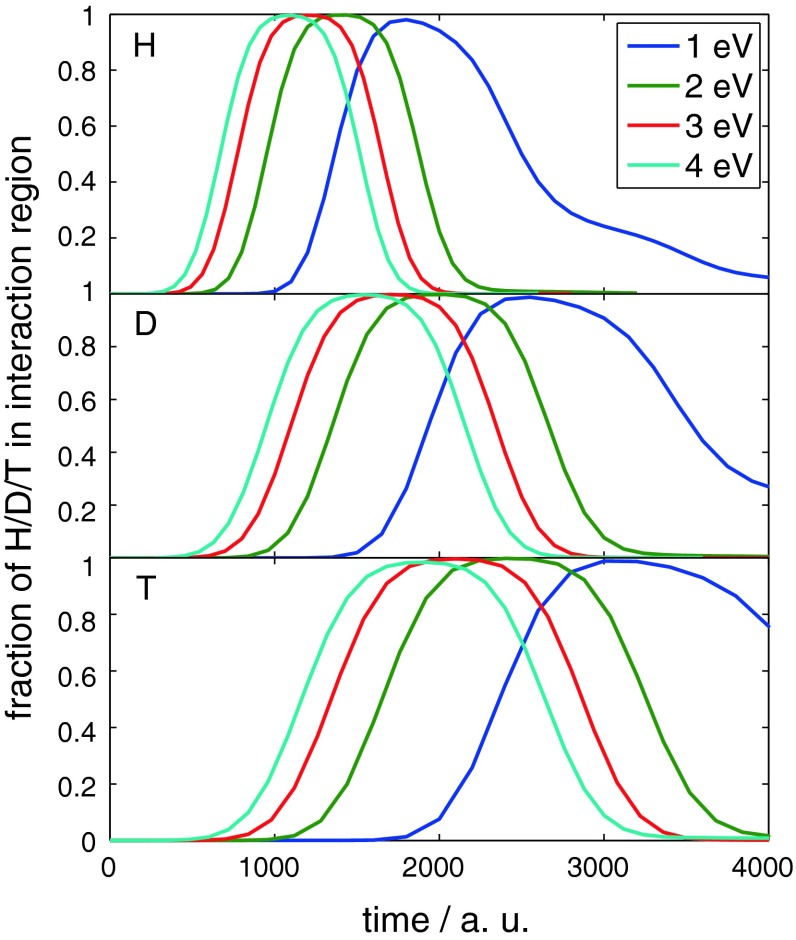
Dwell times in the case of H, D or T scattering at graphite (0001) surfaces for different impact energies

The reason for the difference between He and H/D/T are the various local minima in the H/D/T-graphite potential. This is clearly seen in the plots of the dwell time for hydrogen, deuterium and tritium in Fig. 6. For an energy of 1 eV, the dwell time exhibits a long tail which is a consequence of partial reflection when the wave packet passes through the minima in front of the steep repulsive increase of the potential. Thus, the wave packet is first reflected by the potential wall and then by the edges of the minima, parts of it several times. Hence, the dwell time decreases significantly slower than in the case of He in agreement with an earlier wave packet study focusing on impact energies in a range of 0.1–0.9 eV [31]. At higher energies, the dwell time becomes more Gaussian-shaped as the influence of these features of the H/D/T-graphite potential become less significant in agreement with physical intuition, because as energies become higher the importance of quantum dynamic effects diminishes. Nevertheless, the results at higher energies are in agreement also with a recent investigation of the sticking coefficients of H/D/T on graphite which have been shown to decrease with increasing impact energy [47]. These features as well as the scaling with the square root of the impinging particle’s mass are well reproduced in Fig. 6. The dwell time at 2 eV approaches zero still slower as in the case of He reflection, but the difference is much smaller than at 1 eV. The dwell time curve for 3 eV is already Gaussian-shaped. The curve for the impact energy of 4 eV again exhibits a small tail. This could be due to the fact that the classical permeation energy at bridge site is 4.5 eV and thus is only slightly higher than the impact energy. This subsequently increases the possibility of encountering small potential walls when the wave packet is reflected into regions of higher energy.

**Fig. 7:**
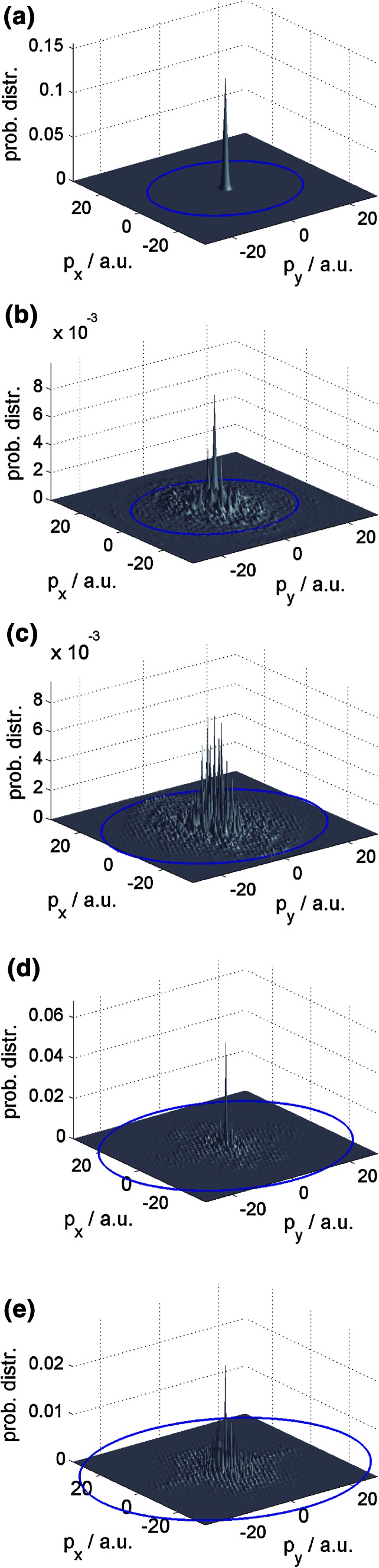
Projection onto the *xy*-plane of the probability distribution in momentum space for an impact of *T* at the bridge site. **a** Initial wave packet. **b** Final probability distribution after reflection for an impact energy of 1 eV. **c** Final distribution after reflection for 2 eV. **d** Final distribution after reflection for 3 eV. **e** Final distribution after reflection for 4 eV. Ewald’s circle is indicated as a *blue line*

Inspection of the projected momentum space probability distributions (Fig. 7) illuminates this. For an impact energy of 1 eV, the different scattering channels extend far to the boundary of Ewald’s circle corresponding to the complex situation of multiple re-reflection at the several potential walls arising from the small-scale structures in the potential. When the impact energy is increased, the scattering channels are well located in the central region of Ewald’s circle, that is the influence of the small scale structures vanishes. For an impact energy of 4 eV, one observes that the probability distribution becomes fuzzy for the reasons discussed above. Since the probability distribution is more centered and Ewald’s circle is larger as the impact energy increases, the overall reflection angle becomes smaller. This explains why the reflection patterns become more concise and compact as the energy is increased, see the discussion of Fig. 5.

### Discussion

We observe a substantial difference between the reflective scattering of He and H/D/T at graphite (0001) surfaces. Whereas He is deflected from the boundary of the hexagonal aromatic cycles constituting the graphite surface toward their centers, the opposite is the case for H/D/T. Though this can be expected as hydrogen more likely reacts with carbon than He, our method provides a microscopic view on the atom-surface scattering process which can be related to several observations from earlier MD simulations [11, 12, 21]. First the role of swift chemical sputtering [11] of carbon-based materials by hydrogen isotopes is significantly emphasized by our findings. They show that hydrogen is very likely to interact with the surface predominantly in this relevant region of the aromatic cycle, especially in those low energy regimes in which chemical sputtering takes place. Second, the non-significance on the sputtering yield at low energies (<10 eV) of small amounts of noble gases mixed into the bombarding species [12] is easily explained. It turns out that He is likely to be deflected toward the centers of the aromatic rings, which means that swift chemical sputtering is not applicable to this situation. In addition, the energy that a He atom might transfer is spread over a larger amount of carbon atoms and bonds such that at low energies He does not contribute much to the sputtering. Since He is prototypical for the chemical behavior of other noble gases, one can directly apply these results to these cases. Third, our results serve as a simple explanatory tool for the graphite peeling process [21], that is the observation that graphite layers are sputtered off one by one. Since hydrogen is deflected toward the boundary of the aromatic cycles, it is unlikely to go through the first layer at low energies without disturbing at least the surface layer via adsorption or bond breaking mechanisms. Thus, the second layer cannot effectively be sputtered until the degradation of the first layer has sufficiently advanced such that hydrogen can go through without being deflected toward the boundary.

## Conclusion

We present a 3D time-dependent wave packet simulation to investigate elastic scattering of tritium and helium on graphite (0001) surfaces. We gain some insight into the physics underlying important mechanisms for chemical erosion of carbon-based materials exposed to hot plasma or gas. In particular, the importance of swift chemical sputtering [11] for the chemical erosion of carbon-based materials is underlined, and a first-principle explanation for observed graphite peeling during hydrogen bombardment [21] is given.

## Electronic supplementary material

Below is the link to the electronic supplementary material.

Supplementary material 1 (MPG 183 kb)

Supplementary material 2 (MPG 194 kb)

Supplementary material 3 (MPG 205 kb)

Supplementary material 4 (MPG 206 kb)

Supplementary material 5 (MPG 187 kb)

Supplementary material 6 (MPG 194 kb)

Supplementary material 7 (MPG 201 kb)

Supplementary material 8 (MPG 204 kb)

Supplementary material 9 (MPG 188 kb)

Supplementary material 10 (MPG 206 kb)

Supplementary material 11 (MPG 201 kb)

Supplementary material 12 (MPG 195 kb)

Supplementary material 13 (TAR 2499 kb)
